# Development and validation of dysphagia knowledge and self-reported competence questionnaire for nurses

**DOI:** 10.1371/journal.pone.0358250

**Published:** 2026-09-15

**Authors:** Raffa Mubeen, Asghar Khan, Humaira Shamim Kiyani, Ayesha Kamal Butt, Hans Bogaardt, Sadaf Noveen

**Affiliations:** 1 Faculty of Rehabilitation and Allied Health Science, Riphah InternationalUniversity, Islamabad, Pakistan; 2 Manchester Metropolitan University, Manchester, United Kingdom; 3 Department of Speech Pathology, School of Primary and Allied Health Care, Monash University, Melbourne, Australia; 4 National Institute of Rehabilitation Medicine, Islamabad, Pakistan; Ankara University, TÜRKIYE

## Abstract

**Background:**

Dysphagia is a common condition across healthcare settings and is associated with serious complications. Nurses play a key role in early identification and safe management. However, substantial gaps exist in nurses’ knowledge and competence. The scope of existing dysphagia questionnaires is limited, and most are designed for specialised populations. This study aimed to develop and validate a comprehensive tool to assess nurses’ knowledge and perceived competence in dysphagia care.

**Methods:**

The Dysphagia Knowledge and Self-reported Competence Questionnaire for Nurses (DKSCQ-N) was developed and validated in two phases. Phase 1 included item generation through literature review, mapping items across six domains, and expert evaluation using a Table of Specification and the Content Validity Index. Cognitive interviews with nurses were conducted to assess clarity, relevance and usability. In Phase 2, data were collected from 537 nurses from hospitals across Pakistan to establish reliability and construct validity. Reliability was assessed using internal consistency and test-retest reliability. Validity was assessed using confirmatory factor analysis and convergent validity.

**Results:**

Expert review demonstrated strong content validity (S-CVI = 0.95). Minor revisions were made after cognitive interviews to improve clarity. Confirmatory factor analysis supported a 21-item, two-factor model with acceptable fit (χ^2^/df = 4.062, CFI = 0.93, TLI = 0.921, RMSEA = 0.076, GFI = 0.88). Both factors demonstrated good convergent and discriminant validity. Significant correlation between the signs and symptoms, complication and management domains of DKSCQ-N and Dysphagia knowledge Scale supported convergent validity. Internal consistency was high (Cronbach’s α = 0.906) and test-retest reliability indicated excellent stability over two-weeks period (ICC = 0.829).

**Conclusion:**

The DKSCQ-N is a valid and reliable instrument to assess dysphagia knowledge and self-reported competence of nurses. It provides a comprehensive and contextually relevant measure that can help identify learning needs and assess training outcomes in hospital settings.

## Introduction

Dysphagia, also termed as swallowing difficulty, is a common clinical condition observed across healthcare settings [1–3]. It is associated with serious complications such as malnutrition, dehydration, aspiration pneumonia, and death [4]. The impact of dysphagia is not limited to physiological issues alone and also includes psychological challenges, such as withdrawal of the patient from society due to anxiety associated with mealtime [5]. Consequently, it affects the overall quality of life of individuals experiencing swallowing difficulty [6].

Prevalence of dysphagia is estimated at 43.8% globally [7], with reported rates of 36.5% in hospital setting, 42.5% in rehabilitation setting, and 50.2% in nursing homes [3]. A hospital-based study in Pakistan reported a rate of 53% in post-stroke patients [8]. Its high prevalence and associated risks make early detection and prompt management essential [9,10]. As nurses spend more time with patients for ongoing care, they are often the first to observe signs of swallowing difficulty [11,12]. Therefore, nurses must be able to recognise signs and symptoms of dysphagia to support early identification, timely referral, and safer patient care [13].

The complications associated with dysphagia highlight the need for interdisciplinary management [14]. Nurses are key members of this team and contribute to both identification and ongoing management of the condition [15]. Appropriate knowledge and competence related to screening and management of dysphagia are essential. However, evidence suggests substantial gaps in nurses’ dysphagia knowledge and skills [16,17], which compromises the quality of dysphagia care [11]. Nurses working in all levels of healthcare demonstrated moderate knowledge regarding poststroke dysphagia [16]. Similarly, neurological nurses scored low on knowledge as compared to their scores in attitude and practice domains [18]. Studies conducted in Pakistan [19] and Namibia [20] highlighted the lack of knowledge among nurses working in hospital settings. Furthermore, nurses working in intensive care units [21] and geriatric settings [22] were found to have insufficient knowledge in this area.

A scarcity of existing questionnaires to assess nurses’ knowledge of dysphagia management led Rhoda et al to develop a new questionnaire [20]. Additional tools have targeted geriatric nurses [22], post-surgical care [23], or neurological settings [18]. However, these tools are limited to specific clinical populations and do not capture the full range of knowledge and perceived competence required in general hospital practice. Moreover, they fail to address all areas of knowledge and perceived competence necessary for identification and management of this condition in hospital settings.

There is therefore a need for a comprehensive, validated instrument that can assess baseline dysphagia knowledge and perceived competence among nurses, identify educational needs and evaluate training outcomes. The aim of this study was to develop and validate the Dysphagia Knowledge and Self-reported Competence Questionnaire for Nurses (DKSCQ-N) using a multi-phase approach incorporating expert review, cognitive interviewing and psychometric testing.

## Materials and methods

### Study design and settings

**Fig 1:**
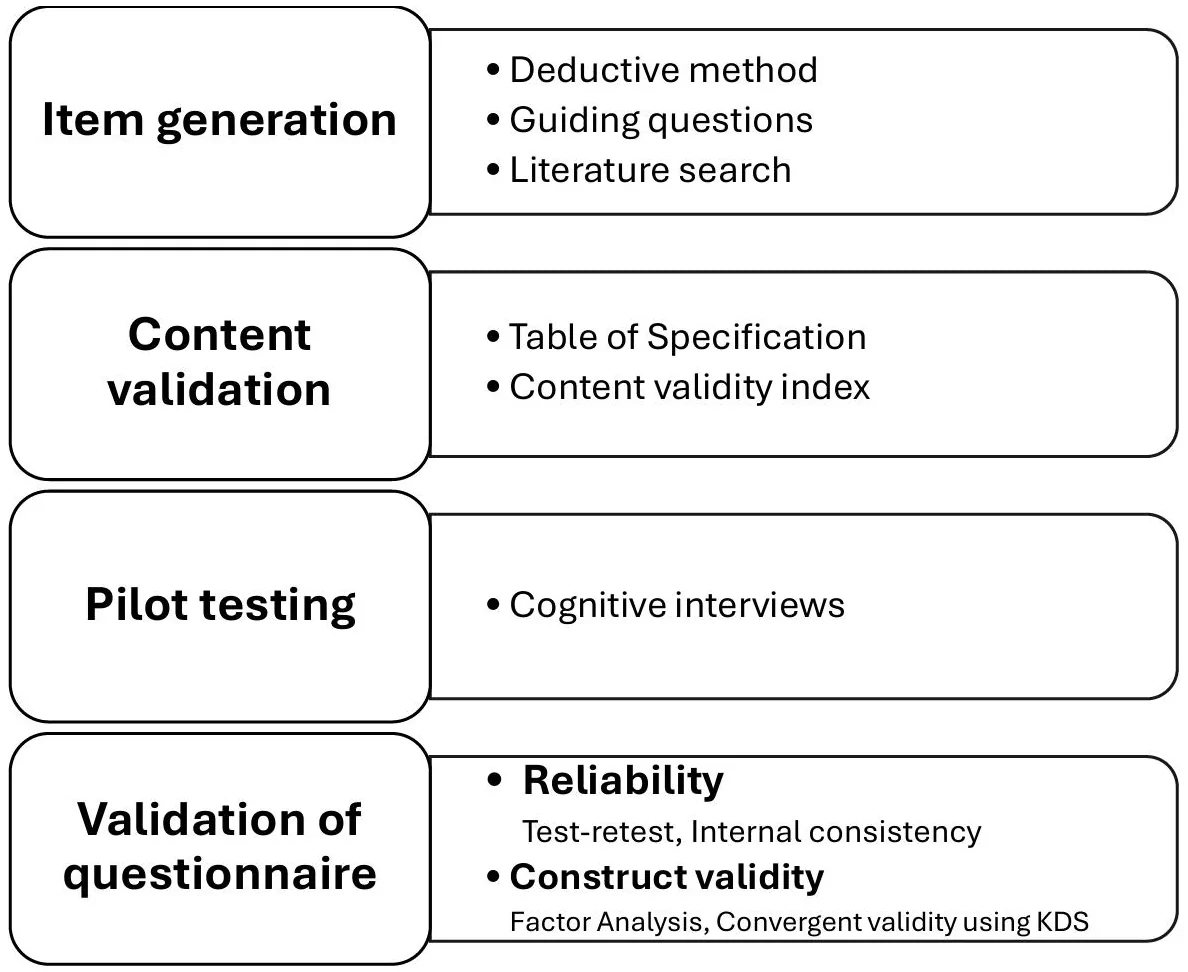
Development and validation process.

The tool was developed and validated in two phases. Phase 1 was concerned with the item generation and content validation to assess if the items adequately measure the domain of interest. The second phase aimed to establish reliability and construct validity of the questionnaire (Fig 1). The study was approved by the Research Ethical Committee of Riphah International University with approval number Riphah/RCRS/REC/13106 dated 09-02-2021. Written informed consent was obtained from all participants, and confidentiality was ensured by assigning numerical codes. Data collection was conducted from 1 May 2022 to 30 June 2022.

### Phase 1: Item generation and content validation

Guiding questions were formulated to facilitate focused search of literature for development of questionnaire [24]. The first question focused on the knowledge nurses should have to deal with patients with dysphagia. The second question focused on the competencies required by nurses to care for patients with dysphagia. Knowledge was defined as understanding of dysphagia-related facts and principles, including anatomy and physiology, risk factors, signs and symptoms, and complications. Self-reported dysphagia care competence was defined as respondents’ judgement of appropriate clinical actions during dysphagia screening and management.

A review of existing literature was conducted to identify key domains related to dysphagia knowledge and competence. Based on this review, six domains were identified: anatomy and physiology, risk factors, signs and symptoms, complications, screening and management. The first four domains contributed to the knowledge construct, while screening and management focused on clinical decision-making during dysphagia care. These domains were used to ensure adequate content coverage and were not intended to represent six separate latent constructs. Using a deductive approach [25], 19 items were generated and aligned with identified domains. The intended use of six domains was to ensure adequate content coverage.

Content validation was conducted using two methods: a) Table of Specification b) Content Validity Index (CVI). Table of specification is a method that aligns a set of items or tasks with a set of concepts aimed for assessment [26]. Domains were listed in columns and items in rows. Five experienced speech language pathologists with clinical and academic background in dysphagia assessment and management were contacted via zoom meeting or face to face meeting as per their convenience. These experts assessed the alignment of items with predefined domains by marking X in the corresponding column. Additionally, they were asked to estimate whether each domain had a sufficient number of items by rating the adequacy of item coverage on a scale from 0 to 100%. They also gave suggestions/ remarks regarding addition, deletion, or revision of some items. The tool was reviewed and revised in light of these suggestions and percentages of each column. The questionnaire was expanded to 25 items after content validation through a table of specification.

The revised version was further evaluated for content validation through content validity index [27]. Seven speech language pathologists with expertise in dysphagia were purposively selected to assess the relevance of each item on a 4-point relevance scale where 1 mean not relevant and 4 mean very relevant. Content validity index was calculated, and 24 items were finalized based on these results.

### Data analysis (Phase 1)

Item to domain alignment and sufficiency of number of items for each domain were calculated from table of specification. The percentage of expert agreement on item-to-domain alignment was calculated using the following formula:


$$\begin{array}{c} (Numberofexpertswhomarkedanitemasaligned/Totalnumberofexperts)\times 100 \end{array}$$

A threshold of 80% was used to include items in a specific domain. Mean sufficiency score for each domain was calculated to determine whether the number of items adequately represented each domain. Domains with mean sufficiency score above 80 were considered to be well represented. Expert qualitative feedback was analyzed to refine items through revision, removal, addition or rephrasing [28].

Both the item level and Scale level Content Validity Index were calculated to assess content validity. Ratings of 3 and 4 were recorded as 1, while ratings of 1and 2 were recorded as 0 [29]. The item level content validity index (I-CVI) was calculated by dividing the number of experts who rated items on rating of 3 or 4 by the number of experts. A threshold of 0.83 was used for the acceptance of item. The S-CVI was calculated by averaging I-CVI of all items. Items falling below threshold were revised, removed or remained based on threshold.

### Pilot testing

The initial version of scale was pretested using cognitive interviews to establish clarity, relevance and usability of the questionnaire for the target population [25]. A total of 15 nurses, both with and without dysphagia training, took part in cognitive interviews that employed verbal probing methods [30] to elicit comprehensive feedback. Participants responses were coded under predefined categories of clarity, relevance and usability issues. The frequency of reported problems was used to identify items which need to be revised or removed. Feedback of participants was considered while revising any specific item.

### Phase 2: validation of the questionnaire

#### Participants, settings, and procedure.

A cross-sectional study design was employed to collect data from nurses for validation of Dysphagia knowledge and self-reported competence questionnaire-Nurses (DKSCQ-N). Nurses with a minimum of one year of clinical experience and working in hospitals across various cities in Pakistan, were included. Nurses working exclusively in gynecology, dermatology, administrative units, and undergraduate nurses were excluded as these roles do not typically involve routine dysphagia screening and management.

A larger sample size is always preferable, as it helps to reduce measurement errors. A sample of 500 is considered ‘good’ according to Comrey and Lee’ guidelines [31]. To meet this requirement, the questionnaire was distributed to 550 nurses. Reliability of the DKSCQ-N was assessed through internal consistency and test-retest reliability. The questionnaire was administered to 46 nurses on two occasions, with a two-week interval between two administrations, to assess test-retest reliability.

The printed version of the DKSCQ-N was used for in-person data collection. The questionnaire consisted of 21 items rated on an 11-point scale (0–10). The knowledge section used a graded response format. Each response had a predetermined correct direction. Several items included both correct responses and incorrect distractors, which were rated separately. Higher scores were expected for correct responses, while distractors were reverse scored. This format allowed respondents to consider each option separately and was intended to provided information about partial knowledge and misconceptions [32,33]. An 11-point rating scale was selected to maintain a consistent response format, retain greater response variation and allow finer differentiation between responses [34,35]. Items covered the domains of anatomy and physiology, risk factors, signs and symptoms, complications, screening and management. Scores for each domain were converted into percentage to ensure standardized scale from 0 to 100.

Construct validity was assessed through Confirmatory Factor Analysis (CFA) and convergent validity. Convergent validity was assessed in two ways. First, CFA-based evidence was used for both constructs through standardized factor loadings and AVE. Second, Pearson correlation was used to examine the relationship between DKSCQ-N domains and KDS. Total 418 participants completed both DKSCQ-N and KDS developed by Andrea Pickle [20]. The correlation between these tools was interpreted as external convergent evidence mainly for the knowledge domain. Therefore, findings related to the self-reported dysphagia care competence domain were interpreted cautiously.

### Data analysis (Phase 2)

Data were analyzed using SPSS version 26 and AMOS version 23. Internal consistency was assessed using Cronbach’s Alpha, and Intraclass Correlation coefficient was used to determine test-retest reliability. Composite Reliability (CR) was calculated using CFA to complement Cronbach’s alpha.

CFA was conducted to test the two-factor structure of the questionnaire. Knowledge items were expected to load on one factor, while self-reported dysphagia care competence items were expected to load on the second factor. This structure reflected the predefined grouping of the six content domains under the two broader constructs. CFA model fit was evaluated using a combination of absolute, incremental, and parsimonious fit indices. Absolute fit indices included Goodness of Fit Index (GFI) and Root Mean Square Error of Approximation (RMSEA). Incremental fit was assessed using Comparative Fit Index (CFI) and Tucker-Lewis Index (TLI). Parsimonious fit was assessed using Chi-square to degree of freedom (χ²/df). For assessing normality, values within ±2 for skewness and ±7 for kurtosis were considered acceptable, as the sample size was greater than 300 [36]. A GFI ≥ 0.90 indicated a good fit, and 0.85 was considered acceptable. RMSEA < 0.05 reflects good fit, and < 0.08 was acceptable. CFI and TLI values ≥ 0.90 were considered acceptable and χ²/df < 5.0 is considered acceptable [37].

Convergent and discriminant validity were assessed after model fit. For convergent validity, Average Variance Extracted (AVE) greater than 0.50 and standardized factor loadings above 0.50 were considered acceptable [38]. Discriminant validity was considered adequate if Maximum Shared Variance was less than Average Variance Extracted for each construct [39], and if the square root of AVE was greater than the inter-construct correlations [40]. Pearson correlation coefficient was used to determine the relationship between subdomains of DKSCQ-N and KDS.

## Results

### Content validity

#### Table of Specification.

Based on feedback from 5 experts using a Table of Specification across six key domains, the alignment of items with the predefined domains exhibits varying levels of agreement. Item 1 received 100% expert agreement in the anatomy and physiology domain. In the risk factor domain, item 2 achieved 80% expert agreement. For signs and symptoms domain, items 3,4, and 5 received agreement levels of 60%,100%, and 80% respectively. In the complication domain, item 5 received 100% agreement, and items 3 and 6 received 80% and 100%, confirming their inclusion in this domain. The screening domain showed high expert agreement, with items 7–15 scoring between 80% and 100%. Similarly, items 16–19 in the management domain received expert agreement ranging from 80 to 100%.

The overall mean sufficiency score was calculated for each domain. The mean score for Anatomy and Physiology domain was 48.4, with individual ratings ranging from 2% to 80%. This suggests that the domain was underrepresented, and three raters suggested adding additional items. The Risk Factors domain received a score of 75%, with individual scores ranging from 45% to 90%. Despite this variation, no additional items were recommended by raters. The Signs and Symptoms, Complications, Screening and Management domains had mean scores of 80.8%, 84.2%, 88.6%, and 83% respective. Therefore, indicating adequate representation. Five new items were added after incorporating expert feedback, increasing the tool to 25 items.

Revisions were made after expert feedback to improve clarity and accuracy. Some terms were replaced, such as changing “oropharyngeal dysphagia” to “swallowing difficulties”. The wording of some items was also refined for better clarity. Irrelevant terms were removed, while clinically relevant terms such as “vomiting” and weight loss” were added. A few items were reworded for consistency, and some terminology was adjusted (e.g., replacing “health” with “medical”).

### Content validity index (CVI)

Out of 25 items, 18 obtained an I-CVI of 1, showing strong agreement among experts. Five items with an I-CVI of .83 were also retained. One item with an I-CVI of 0 was removed because it was not considered relevant. Another item with an I-CVI of 0.67 was revised following expert feedback. The initial S-CVI was 0.91, which increased to 0.95 after removing the item with a zero CVI score.

### Cognitive Debriefing

Cognitive interviews were conducted to assess the clarity, relevance, and usability of the questionnaire. Most nurses reported no major issues. However, three nurses noted concerns related to clarity, four identified relevance related issues, and one reported a usability problem. Clarity concerns were addressed by simplifying technical terms and reducing sentences length. Relevance concerns were mainly related to referral items and distractor options. Distractors were intentionally retained to assess nurses’ ability to recognise incorrect responses. The referral item was also retained because it reflects an important component of dysphagia management. Minor formatting issues were corrected to improve usability.

### Demographics

A total of 548 nurses completed the questionnaire. However, 11 responses were excluded because of incomplete data. The final sample included 537 nurses. Demographic characteristics are presented in Table 1.

**Table 1:** Demographic characteristics of participants (N = 537).

Variable		N(%)
**Gender**	Male	95(17.7)
	Female	442(82.3)
**Qualification**	BSN^a^	245(45.6)
	Diploma	284(52.9)
	MSc^b^	4(.7)
	PhD^c^	4(.7)
**Age**	21-30	185(34.5)
	31-40	325(60.5)
	41-50	20(3.7)
	51-60	7(1.3)
**Experience (Years)**	1-3	98(18.2)
	4-7	195(36.3)
	8-10	150(27.9)
	More than 10	94(17.5)

^a^BSN = Bachelor of Science in Nursing.

^b^MSc = Master of Science.

^c^PhD = Doctor of Philosophy.

### Construct Validity

#### Confirmatory Factor Analysis.

CFA was conducted to verify the two-factor structure of the Dysphagia Knowledge and Self-reported Competence Questionnaire for Nurses. The model, comprising knowledge and self-reported competence factors, was consistent with the initial conceptual structure of the questionnaire. The first four content domains represented knowledge construct, while screening and management represented self-reported dysphagia care competence construct. Thus, the CFA evaluated the predefined two-factor conceptual model rather than a six factor structure. Skewness values ranged from −0.914 to 0.775, while kurtosis values ranged from −1.215 to1.5. All values were within the acceptable range of skewness and kurtosis thus confirming the assumption of univariate normality.

The initial model demonstrated poor fit (χ^2^/df = 5.075, CFI = 0.88, TLI = 0.87, RMSEA = 0.087, GFI = 0.81), indicating a need for refinement. Two anatomy-related items and one screening item with standardised factor loadings below 0.50 were removed. The final refined model, consisting of nine knowledge items and 12 self-reported competence items, showed improved fit (χ^2^/df = 4.062, CFI = 0.93, TLI = 0.921, RMSEA = 0.076, GFI = 0.88). All retained items loaded significantly on their respective factors with standard factors loading ranging from 0.56 to 0.90. The correlation between the two latent constructs was weak (r = 0.13), indicating that knowledge and self-reported competence represent distinct but related domains within the tool (Table 2).

**Table 2:** Model fit indices for the initial and refined two-factor CFA models of the DKSCQ-N.

Model	χ^2^/df^a^	CFI^b^	TLI^c^	RMSEA^d^	GFI^e^
**2 Factors, 24 Items**	5.075	0.881	0.869	0.087	0.814
**2 Factors, 21 Items**	4.062	0.93	0.921	0.076	0.88

^a^χ^2^/df = Chi-square divided by degrees of freedom.

^b^CFI = Comparative Fit Index.

^c^TLI = Tucker-Lewis Index.

^d^RMSEA = Root Mean Square Error of Approximation.

^e^GFI = Goodness of Fit Index

### Convergent and Discriminant Validity

As shown in Table 3, all items had standardised factor loadings above 0.50, satisfying the minimum requirement for item reliability. The Average Variance Extracted (AVE) values were 0.56 for the knowledge factor and 0.60 for self-reported competence factor, both exceeding the threshold of 0.50. These values supported convergent validity of both constructs. Discriminant validity was supported, as the square root of AVE exceeded the inter-construct correlation for both factors, and MSV values were lower than the AVE values. Composite Reliability (CR) values were above 0.70 for both knowledge (CR = 0.91) and self-reported competence (CR = 0.94), demonstrating strong internal consistency.

**Table 3:** Confirmatory Factor Analysis of DKSCQ-N.

Factors	No. of items	Range of standardized factor Loadings (λ)	CR^a^	AVE^b^	MSV^c^	√AVE^a^
Knowledge	9	0.56-0.87	0.91	0.56	0.017	0.748
Self -reported Competence	12	0.56-0.90	0.94	0.60	0.017	0.772

^a^CR = Composite Reliability.

^b^AVE = Average Variance Extracted.

^c^MSV = Maximum Shared Variance.

### Construct Validity: Convergent Validity Using External Tool

After confirming normality for both the DKSCQ-N and KDS scores, Pearson correlation was applied to assess the relationship between the existing and newly developed tool. The results presented in Table 4 indicate a strong and statistically significant correlation across the corresponding subdomains. These findings provide evidence for knowledge domain, as KDS is primarily a knowledge-based scale. All correlations were significant at p < 0.001.

**Table 4:** Convergent Validity between DKSCQ-N and KDS (N = 418).

KDS^a^ × DKSCQ-N^b^	R	Sig.
Signs and Symptoms	0.799^**^	< 0.001
Complications	0.896^**^	< 0.001
Management	0.715^**^	< 0.001

**. Correlation is significant at the < 0.01 (2-tailed).

^a^KDS = Knowledge of Dysphagia Scale,

^b^DKSCQ-N = Dysphagia Knowledge and Self-reported Competence Questionnaire for Nurses.

### Reliability

#### Test-Retest and Internal consistency.

The test-retest reliability was assessed using the Intraclass Correlation Coefficient (ICC). The ICC value of 0.829 (95% CI:0.711–0.902, p<0.001) indicated excellent stability of the tool over a two-week interval. Internal consistency was evaluated using Cronbach’s Alpha. The final 21-item scale yielded a Cronbach’s alpha of 0.906. This demonstrates excellent internal consistency and strong interrelatedness among items. Composite reliability (CR) values further supported reliability, with both the knowledge and self-reported competence factors exceeding the recommended thresholds.

## Discussion

This study focused on the development and validation of a new questionnaire to assess knowledge and self-reported competence of nurses in dysphagia management. The validation was carried out using established steps commonly followed in health measurement tool development. The study provides evidence for reliability and validity of DKSCQ-N [41].

The questionnaire was developed using established steps commonly followed in health measurement tool development. It started with domain identification and item generation. A Table of Specification (ToS) was then used to check whether the six domains were adequately represented. This helped reduce construct underrepresentation and supported validity of the tool [28]. The items were further reviewed by experts using the Content Validity Index (CVI). Most items showed strong agreement among experts. A few items met the minimum acceptable value of 0.83 and were therefore retained [27,29,42]. One item was removed as experts considered it less relevant. Another item with moderate agreement (I-CVI = 0.67) was revised based on expert suggestions because it addressed an important aspect of dysphagia care [29]. After these changes, the scale-level CVI (S-CVI) improved from 0.91 to 0.95, indicating excellent content validity of the final tool.

Cognitive interviews helped to check how nurses understood and responded to the items in the DKSCQ-N. Most participants did not report any concern. However, a few issues were identified related to clarity, relevance, and usability of items. These issues were addressed by simplifying technical wording and improving the format of the questionnaire. Referral-related items and distractors were intentionally retained because they reflected common clinical decision-making situations in practice. These refinements improved the clarity and made the items easier to understand. This process was in line with recommended practices for cognitive interviewing [30] and health scale development [25]. By adding cognitive interviewing as part of pilot testing, the present study ensured that the questionnaire reflected the views of nurses, not only expert opinion.

The two-factor structure, including knowledge and self-reported dysphagia care competence, was supported by both statistical and clinical perspectives. This structure reflected the predefined grouping of the six content domains under the two broader constructs of knowledge and self-reported competence. The model fit values were within acceptable ranges. The validity findings were consistent with current recommendations for confirmatory factor analysis and reporting of reliability and validity [37,39,40]. Previous studies have mainly examined nurses’ knowledge of dysphagia. Findings from these studies suggest that nurses have moderate knowledge, particularly regarding the identification and management of dysphagia [16,17].

This shows that assessing knowledge alone may not be sufficient. The second factor represented respondents’ judgement of appropriate clinical actions during dysphagia screening and management. Activity-based items have also been used to assess nursing competence, including patient assessment and clinical decision-making [43]. Clinical reasoning has also been assessed as a nursing competency through a validated questionnaire-based scale [44]. Therefore, this factor represents self-reported dysphagia care competence related to clinical judgement and decision-making. It should not be interpreted as directly observed clinical performance.

These outcomes are consistent with recent psychometric guidelines, which recommend using different forms of validity evidence [37,39]. Similar CFA-based approaches have also been used in nursing questionnaire validation studies to assess convergent and discriminant validity [45,46]. Findings of the present study support the validity and reliability of DKSCQ-N.

Reliability of the DKSCQ-N was supported by both internal consistency and test-retest findings. Internal consistency was excellent and was above the commonly accepted threshold of 0.70. This indicates that the items were well related to each other [25]. Similar reliability values have been reported in other nursing questionnaires related to dysphagia and clinical competencies, with values range from 0.80 to 0.95 [18,22,46]. Test-retest reliability was also strong and showed stability of the toll over time. The ICC value was above the recommended cutoffs of 0.75 for good reliability and 0.80 for excellent reproducibility [47]. These findings suggest that DKSCQ-N can be used for baseline assessment and for measuring change after educational interventions.

Several questionnaires have been developed to assess nurses’ knowledge of dysphagia. However, most of these tools focus on specific patient groups, such as stroke, geriatric, or post-surgical patients [18,20,22,23]. They do not fully cover the broader role of nurses in dysphagia care. In the present study, DKSCQ-N was developed and validated to assess both knowledge and self-reported dysphagia care competence. Previous KAP questionnaires covered selected aspects of dysphagia. DKSCQ-N was designed to capture both knowledge and self-reported competence across a broader range of dysphagia related responsibilities encountered by nurses in hospital settings.

A major strength of this study is its multi-step validation process. The use of ToS, CVI, and cognitive interviewing made the questionnaire psychometrically sound and clinically relevant. This approach is recommended as best practice in scale development [25]. The inclusion of correct responses and distractors required participants to consider each option separately. This helped assess whether they could identify correct clinical information and reject incorrect information [32,33].

The results indicate that DKSCQ-N can be used for multiple purposes. It can help identify training needs across specific domains and support targeted educational interventions. It can also be used as a pre- and post-test measure in training programs. This is supported by earlier evidence showing that blended learning interventions can improved dysphagia-related knowledge and competence [48]. Future studies may examine whether improvement in DKSCQ-N scores is linked with better patient outcomes.

### Limitations

Despite its strengths, this study has some limitations. Data were collected from nurses working in hospital settings in Pakistan. Therefore, the findings may not be fully generalizable to other countries or healthcare systems. Future studies should validate the DKSCQ-N in other cultural, educational and healthcare contexts before wider use.

The self-reported dysphagia care competence domain was based on nurses’ own ratings. Therefore, it reflects their perceived ability to apply dysphagia care actions and should not be interpreted as directly observed clinical performance. Future studies may examine this domain through simulation-based assessment, direct observation, or clinical competency checklists.

Another limitation is related to the external comparison scale. The KDS mainly assessed dysphagia knowledge. Therefore, its correlation with the DKSCQ-N provides external support mainly for the knowledge domain. The self-reported competence domain was supported through CFA-based validity evidence, factor loadings, and AVE. However, it was not externally validated against an established competence-specific dysphagia tool, as existing tools focus on knowledge or KAP outcomes and no validated competence-related measure was available for comparison.

The knowledge items had a predetermined correct scoring direction. However, the ratings may also reflect respondents’ certainty or response style along with factual knowledge. Future studies should compare the DKSCQ-N knowledge scores with an objective multiple-choice or true/false knowledge measure covering the same content.

## Conclusion

This study developed and validated DKSCQ-N to assess nurses’ knowledge and self-reported dysphagia care competence. The questionnaire showed good validity and reliability. It addresses gaps in existing tools by covering key areas of dysphagia care, including screening, referral and management. The DKSCQ-N can be used to identify training needs and to evaluate changes in knowledge and self-reported competence before and after dysphagia-related training programs.

## Supporting information

S1 File Table of Specification. (DOCX)

S2 File DKSC Questionnaire for Nurses. (DOCX)

S3 File DKSCQ-N Scoring Manual. (DOCX)

S4 File DKSCQ-N Data Entry Sheet. (XLSX)

S5 File Items removed during Confirmatory Factor Analysis. (DOCX)
