# Supplementary material for: Development and validation of dysphagia knowledge and self-reported competence questionnaire for nurses
Source: PLoS One. 2026 Sep 15;21(9):e0358250. doi: 10.1371/journal.pone.0358250 (PMC13577460; doi:10.1371/journal.pone.0358250)
Supplement: Supplementary file 1 [file pone.0358250.s001.docx]

| **Item number** | **Anatomy and physiology** | **Risk factors** | **Sign and symptoms** | **complications** | **Screening** | **Management** |
| --- | --- | --- | --- | --- | --- | --- |
|  |  |  |  |  |  |  |
| 1 | **100** | 0 | 0 | 0 | 0 | 0 |
| 2 | 0 | **80** | 0 | 0 | 40 | 0 |
| 3 | 20 | 40 | 60 | **80** | 0 | 0 |
| 4 | 20 | 20 | **100** | 0 | 20 | 0 |
| 5 | 20 | 20 | **80** | **100** | 40 | 0 |
| 6 | 0 | 0 | 40 | **100** | 0 | 0 |
| 7 | 0 | 0 | 0 | 20 | **80** | 60 |
| 8 | 0 | 0 | 0 | 0 | **100** | 20 |
| 9 | 0 | 0 | 0 | 0 | **100** | 20 |
| 10 | 0 | 0 | 0 | 0 | **80** | 40 |
| 11 | 20 | 20 | 0 | 0 | **100** | 40 |
| 12 | 0 | 20 | 20 | 0 | **80** | 60 |
| 13 | 20 | 20 | 20 | 0 | **80** | 0 |
| 14 | 0 | 0 | 0 | 0 | **100** | 0 |
| 15 | 0 | 0 | 0 | 0 | **100** | 0 |
| 16 | 0 | 0 | 0 | 0 | 0 | **100** |
| 17 | 0 | 0 | 0 | 0 | 20 | **80** |
| 18 | 20 | 0 | 0 | 0 | 0 | **100** |
| 19 | 0 | 0 | 0 | 0 | 0 | **100** |
|  |  |  |  |  |  |  |
