# Supplementary material for: Development and validation of dysphagia knowledge and self-reported competence questionnaire for nurses
Source: PLoS One. 2026 Sep 15;21(9):e0358250. doi: 10.1371/journal.pone.0358250 (PMC13577460; doi:10.1371/journal.pone.0358250)
Supplement: Supplementary file 2 [file pone.0358250.s002.docx]

**DYSPHAGIA KNOWLEDGE AND SELF-REPORTED COMPETENCE QUESTIONNAIRE FOR NURSES**

**Section A: Demographics**

Please tick (✔) or fill in the appropriate response.

**Age:** ____________Years

**Gender:**

☐ Male

☐ Female

Highest Qualification: _________________

Years of clinical experience: ___________________

Current Ward placement: ________________

Previous Ward placements: ____________________

Have you ever managed a patient with dysphagia?

☐ Yes

☐ No

If yes, please indicate the ward/unit where you encountered dysphagia patient.

Have

☐ Yes

☐ No

If yes, please specify the type and duration of training

__________________________

**Section B: Knowledge and skills items**

**Mark following items on scale of 0 to 10**

| **1** | **Select the structures from the following that are related to oropharyngeal phase of swallowing.** | |
| --- | --- | --- |
|  |  | **Strongly Disagree Neutral Strongly Agree** |
| i. | Oral cavity | 0 1 2 3 4 5 6 7 8 9 10 |
| ii. | Soft palate | 0 1 2 3 4 5 6 7 8 9 10 |
| iii. | Nasal cavity | 0 1 2 3 4 5 6 7 8 9 10 |
| iv. | Pharynx | 0 1 2 3 4 5 6 7 8 9 10 |
| v. | Larynx | 0 1 2 3 4 5 6 7 8 9 10 |
| vi. | Trachea | 0 1 2 3 4 5 6 7 8 9 10 |
| vii. | Esophagus | 0 1 2 3 4 5 6 7 8 9 10 |
| viii. | Stomach | 0 1 2 3 4 5 6 7 8 9 10 |

| **2** | **Which of the following stage is related to preparation of food for swallow?** | |
| --- | --- | --- |
|  |  | **Not related at all Neutral Strongly related** |
| i. | Oral preparatory stage | 0 1 2 3 4 5 6 7 8 9 10 |
| ii. | Oral propulsion stage | 0 1 2 3 4 5 6 7 8 9 10 |
| iii. | Pharyngeal stage | 0 1 2 3 4 5 6 7 8 9 10 |
| iv. | Esophageal stage | 0 1 2 3 4 5 6 7 8 9 10 |

| **3** | **Which of the following stage is related to transit of bolus to the esophagus and physiological protective response?** | |
| --- | --- | --- |
|  |  | **Not related at all Neutral Strongly related** |
| i. | Oral preparatory stage | 0 1 2 3 4 5 6 7 8 9 10 |
| ii. | Oral propulsion stage | 0 1 2 3 4 5 6 7 8 9 10 |
| iii. | Pharyngeal stage | 0 1 2 3 4 5 6 7 8 9 10 |
| iv. | Esophageal stage | 0 1 2 3 4 5 6 7 8 9 10 |

| **4** | **Airway must be closed, and food pipe must be open while swallowing.** |
| --- | --- |
|  | **Strongly Disagree Neutral Strongly Agree** |
|  | 0 1 2 3 4 5 6 7 8 9 10 |

| **5** | **Which of the following conditions can cause swallowing difficulties/dysphagia?** | |
| --- | --- | --- |
|  |  | **Strongly Disagree Neutral Strongly Agree** |
| i. | Neurological | 0 1 2 3 4 5 6 7 8 9 10 |
| ii. | Respiratory | 0 1 2 3 4 5 6 7 8 9 10 |
| iii. | Cardiovascular | 0 1 2 3 4 5 6 7 8 9 10 |
| iv. | Autoimmune | 0 1 2 3 4 5 6 7 8 9 10 |
| v. | Head and neck cancer | 0 1 2 3 4 5 6 7 8 9 10 |
| vi. | Surgeries of brain and head/neck region | 0 1 2 3 4 5 6 7 8 9 10 |
| vii. | Substance abuse | 0 1 2 3 4 5 6 7 8 9 10 |
| viii. | Psychogenic | 0 1 2 3 4 5 6 7 8 9 10 |

| **6** | **The clients with swallowing difficulty are at high risk of developing pneumonia.** |
| --- | --- |
|  | **Strongly Disagree Neutral Strongly Agree** |
|  | 0 1 2 3 4 5 6 7 8 9 10 |

| **7** | **Which of the following symptoms are related to swallowing difficulties/dysphagia?** | |
| --- | --- | --- |
|  |  | **Strongly disagree Neutral Strongly agree** |
| i. | Food spillage from lips | 0 1 2 3 4 5 6 7 8 9 10 |
| ii. | Drooling | 0 1 2 3 4 5 6 7 8 9 10 |
| iii. | Stomachache | 0 1 2 3 4 5 6 7 8 9 10 |
| iv. | Nasal regurgitation | 0 1 2 3 4 5 6 7 8 9 10 |
| v. | Pain in ear | 0 1 2 3 4 5 6 7 8 9 10 |
| vi. | Coughing | 0 1 2 3 4 5 6 7 8 9 10 |
| vii. | Choking | 0 1 2 3 4 5 6 7 8 9 10 |
| viii. | Headache | 0 1 2 3 4 5 6 7 8 9 10 |
| ix. | Avoidance of certain food | 0 1 2 3 4 5 6 7 8 9 10 |
| x. | Residue in oral cavity | 0 1 2 3 4 5 6 7 8 9 10 |
| xi. | Diarrhea | 0 1 2 3 4 5 6 7 8 9 10 |
| xii. | Vomiting | 0 1 2 3 4 5 6 7 8 9 10 |
| xiii. | Multiple swallow attempts | 0 1 2 3 4 5 6 7 8 9 10 |

| **8** | **Which of the following problems are associated with swallowing difficulties?** | |
| --- | --- | --- |
|  |  | **Strongly disagree Neutral Strongly Agree** |
| i. | Sleep apnea | 0 1 2 3 4 5 6 7 8 9 10 |
| ii. | Compromised quality of life | 0 1 2 3 4 5 6 7 8 9 10 |
| iii. | Prolonged meal duration | 0 1 2 3 4 5 6 7 8 9 10 |
| iv. | Repeated chest infections | 0 1 2 3 4 5 6 7 8 9 10 |
| v. | Repeated hospital admission and extended stay | 0 1 2 3 4 5 6 7 8 9 10 |
| vi. | Blurred vision | 0 1 2 3 4 5 6 7 8 9 10 |
| vii. | Change in voice | 0 1 2 3 4 5 6 7 8 9 10 |
| viii. | Vasovagal syncope | 0 1 2 3 4 5 6 7 8 9 10 |

| **9** | **Which of the following are complications of swallowing difficulties?** | |
| --- | --- | --- |
|  |  | **Strongly Disagree Neutral Strongly Agree** |
| i. | Ataxia | 0 1 2 3 4 5 6 7 8 9 10 |
| ii. | Mortality | 0 1 2 3 4 5 6 7 8 9 10 |
| iii. | Heart problems | 0 1 2 3 4 5 6 7 8 9 10 |
| iv. | Pneumonia | 0 1 2 3 4 5 6 7 8 9 10 |
| v. | Malnutrition | 0 1 2 3 4 5 6 7 8 9 10 |
| vi. | Dehydration | 0 1 2 3 4 5 6 7 8 9 10 |
| vii. | Weight loss | 0 1 2 3 4 5 6 7 8 9 10 |

| **10** | **A screening tool should be used by nurses to identify patients with possible oropharyngeal dysphagia.** |
| --- | --- |
|  | **Strongly Disagree Neutral Strongly Agree** |
|  | 0 1 2 3 4 5 6 7 8 9 10 |

| **11** | **Only dysphagia trained nurses should screen patients who are at risk of oropharyngeal dysphagia.** |
| --- | --- |
|  | **Strongly Disagree Neutral Strongly Agree** |
|  | 0 1 2 3 4 5 6 7 8 9 10 |

| **12** | **A stroke patient should be kept NPO (not per oral) until screened for risk of swallowing difficulties.** |
| --- | --- |
|  | **Strongly Disagree Neutral Strongly Agree** |
|  | 0 1 2 3 4 5 6 7 8 9 10 |

| **13** | **Screening should be postponed and NPO is recommended if patient cannot be screened due to their cognitive state or medical condition.** |
| --- | --- |
|  | **Strongly Disagree Neutral Strongly Agree** |
|  | 0 1 2 3 4 5 6 7 8 9 10 |

| **14** | **Which of the following factor(s) can impact the ability to eat and drink safely?** | |
| --- | --- | --- |
|  |  | **Strongly Disagree Neutral Strongly Agree** |
| i. | Alertness | 0 1 2 3 4 5 6 7 8 9 10 |
| ii. | Command following | 0 1 2 3 4 5 6 7 8 9 10 |
| iii. | Ability to walk | 0 1 2 3 4 5 6 7 8 9 10 |
| iv. | Gag reflex | 0 1 2 3 4 5 6 7 8 9 10 |
| v. | Ability to speak clearly | 0 1 2 3 4 5 6 7 8 9 10 |

| **15** | **Which of the following liquid(s) is commonly used during bedside screening for swallowing difficulties?** | |
| --- | --- | --- |
|  |  | **Strongly Disagree Neutral Strongly Agree** |
| i. | Water | 0 1 2 3 4 5 6 7 8 9 10 |
| ii. | Tea | 0 1 2 3 4 5 6 7 8 9 10 |
| iii. | Milkshake | 0 1 2 3 4 5 6 7 8 9 10 |

| **16** | **What quantity of liquid can be used to identify patients with swallowing difficulties?** | |
| --- | --- | --- |
|  |  | **Strongly Disagree Neutral Strongly Agree** |
| i. | 5-30mL | 0 1 2 3 4 5 6 7 8 9 10 |
| ii. | 90-100 mL | 0 1 2 3 4 5 6 7 8 9 10 |
| iii. | 200-250mL | 0 1 2 3 4 5 6 7 8 9 10 |

| **17** | **Which of the following come under nursing management when dealing with a patient with oropharyngeal dysphagia?** | |
| --- | --- | --- |
|  |  | **Strongly Disagree Neutral Strongly Agree** |
| i. | Management of posture at mealtime | 0 1 2 3 4 5 6 7 8 9 10 |
| ii. | Recommend compensatory techniques | 0 1 2 3 4 5 6 7 8 9 10 |
| iii. | Verifying diet texture as recommended | 0 1 2 3 4 5 6 7 8 9 10 |
| iv. | Oral care | 0 1 2 3 4 5 6 7 8 9 10 |
| v. | Referral to SLP if status changes | 0 1 2 3 4 5 6 7 8 9 10 |

| **18** | **Who (professional) should a patient be referred to for swallow assessment if swallowing difficulty is identified?** | |
| --- | --- | --- |
|  |  | **Strongly Disagree Neutral Strongly Agree** |
| i. | Dietitian | 0 1 2 3 4 5 6 7 8 9 10 |
| ii. | Gastroenterologist | 0 1 2 3 4 5 6 7 8 9 10 |
| iii. | Speech and language pathologist | 0 1 2 3 4 5 6 7 8 9 10 |

| **19** | **Which posture is mostly recommended for the person diagnosed with oropharyngeal dysphagia while eating?** | |
| --- | --- | --- |
|  |  | **Strongly Disagree Neutral Strongly Agree** |
| i. | Lying on back | 0 1 2 3 4 5 6 7 8 9 10 |
| ii. | Sitting at 45º | 0 1 2 3 4 5 6 7 8 9 10 |
| iii. | Sitting at 90º | 0 1 2 3 4 5 6 7 8 9 10 |

| **20** | **Which diet might be prescribed by the speech and language pathologist for patients with swallowing difficulties?** | |
| --- | --- | --- |
|  |  | **Strongly Disagree Neutral Strongly Agree** |
| i. | Normal hospital diet | 0 1 2 3 4 5 6 7 8 9 10 |
| ii. | Thin liquids | 0 1 2 3 4 5 6 7 8 9 10 |
| iii. | Thick liquids | 0 1 2 3 4 5 6 7 8 9 10 |
| iv. | Solid food items | 0 1 2 3 4 5 6 7 8 9 10 |
| v. | Pureed items | 0 1 2 3 4 5 6 7 8 9 10 |

| **21** | **Dysphagia trained nurses can clearly identify food items according to diet levels.** |
| --- | --- |
|  | **Strongly Disagree Neutral Strongly Agree** |
|  | 0 1 2 3 4 5 6 7 8 9 10 |
