# Supplementary material for: Development and validation of dysphagia knowledge and self-reported competence questionnaire for nurses
Source: PLoS One. 2026 Sep 15;21(9):e0358250. doi: 10.1371/journal.pone.0358250 (PMC13577460; doi:10.1371/journal.pone.0358250)
Supplement: Supplementary file 3 [file pone.0358250.s003.docx]

**Dysphagia Knowledge and Self-reported Competence Questionnaire for Nurses- Scoring Manual**

The tool consists of 21 items grouped into two main domains: knowledge and Self-reported competence. Item numbers 1 to 9 are concerned with knowledge, and item numbers 10 to 21 are concerned with Self-reported competence. Each item has sub-items, thus score of each item is different. Each sub-item is scored on a 0-10 scale, where 0 represents strongly disagree and 10 represents strongly agree. Higher score reflects better knowledge or Self-reported competence.

There are a few sub-items that act as distractors and reverse scored. These distractors are designed to check accuracy. A lower score on these items indicates higher knowledge. Blank or unanswered sub-items are scored as 0. All sub items scores are summed to calculate each item score after applying reverse scoring where needed. Domain totals are obtained by adding the scores of all items of that domain. The overall score represents the sum of knowledge and Self-reported competence domains.

| Items |  |  |  | Minimum Score range | Maximum Score range |
| --- | --- | --- | --- | --- | --- |
| 1 | Anatomy | Theoretical background/knwoledge | **Knowledge** | 0 | 80 |
| 2 | Anatomy/physiology |  |  | 0 | 40 |
| 3 | Anatomy/physiology |  |  | 0 | 40 |
| 4 | Anatomy |  |  | 0 | 10 |
| 5 | Risk factors |  |  | 0 | 80 |
| 6 | Complications |  |  | 0 | 10 |
| 7 | Sign and symptoms |  |  | 0 | 130 |
| 8 | Complications |  |  | 0 | 80 |
| 9 | Complications |  |  | 0 | 70 |
| 10 | Screening | Screening | **Self-Reported Competence** | 0 | 10 |
| 11 | Screening |  |  | 0 | 10 |
| 12 | Screening |  |  | 0 | 10 |
| 13 | Screening |  |  | 0 | 10 |
| 14 | Screening |  |  | 0 | 50 |
| 15 | Screening |  |  | 0 | 30 |
| 16 | Screening |  |  | 0 | 30 |
| 17 | Management |  |  | 0 | 50 |
| 18 | Management | Management |  | 0 | 30 |
| 19 | Management |  |  | 0 | 30 |
| 20 | Management |  |  | 0 | 50 |
| 21 | Management |  |  | 0 | 10 |

**Maximum Scores**

Knowledge= 540

Self-reported competences= 320

Total= 860

**Reverse Scoring Items**

- Following sub-items are ***reverse scored*** and a strongly disagree indicates correct understanding.

| **Item** | **Reverse-Scored Sub-items** |
| --- | --- |
| **1** | iii (Nasal cavity), vi (Trachea), vii (Esophagus), viii (Stomach) |
| **2** | ii (Oral propulsion), iii (Pharyngeal), iv (Esophageal) |
| **3** | i (Oral preparatory), ii (Oral propulsion), iv (Esophageal) |
| **7** | iii (Stomachache), vi (Pain in ear), viii (Headache), xi (Diarrhea), xii (Vomiting) |
| **8** | i (Sleep apnea), vi (Blurred vision), viii (Vasovagal syncope) |
| **9** | i (Ataxia), iii (Heart problems) |
| **14** | iii (Ability to walk), iv (Gag reflex) |
| **15** | ii (Tea), iii (Milkshake) |
| **16** | iii (200-250 ml) |
| **17** | ii (Recommend compensatory techniques) |
| **18** | i (Dietitian), ii (Gastroenterologist) |
| **19** | i (Lying on back) |

**Steps for Scoring**

1. Ensure all sub-items are marked between 0 and 10.
2. For each reverse-scored sub-item, calculate the adjusted score using:

Adjusted score = 10 - Raw score

Example:

- If a participant gives **8**, the adjusted score becomes **2**.
- If the response is **0**, the adjusted score becomes **10**.
- If the response is **blank**, the adjusted score is **0**.

3. Add all sub-item scores for each item.
4. Sum the item totals within each domain to obtain the domain total score.
5. Add all domain totals to get the overall total score (out of 860).

Overall Total = Knowledge + Self-reported competences

6. Convert to percentage:

$$\text{Percentage}=\frac{\text{Obtained Score}}{\text{Maximum Possible Score}}\times100$$

**Handling Missing Responses**

- If only a few sub-items (up to 10%) within a domain are missing, calculate the average of the answered sub-items in that domain and use this average score for the missing ones.
- If more than 10% of the sub-items in a domain are missing, the domain score should not be calculated.

A conservative threshold of 10% was selected to ensure that domain scores remained representative of respondents’ knowledge and self-reported competence while minimising the influence of imputed values. In a simulation study of a multi-item self-report scale, individual-mean imputation showed near-perfect agreement with complete scores at 10% missingness, while agreement decreased as the amount of missing data increased (Shrive et al., 2006).

**Reference**

1. Shrive, F.M., Stuart, H., Quan, H. *et al.* Dealing with missing data in a multi-question depression scale: a comparison of imputation methods. *BMC Med Res Methodol* **6**, 57 (2006). https://doi.org/10.1186/1471-2288-6-57
