# Supplementary material for: Development and validation of dysphagia knowledge and self-reported competence questionnaire for nurses
Source: PLoS One. 2026 Sep 15;21(9):e0358250. doi: 10.1371/journal.pone.0358250 (PMC13577460; doi:10.1371/journal.pone.0358250)
Supplement: Supplementary file 4 [file pone.0358250.s005.docx]

**S6: Items removed during Confirmatory Factor Analysis**

| **Removed item** | **Original domain** | **Intended factor** | **Standardized factor loading** | **Removal criterion** |
| --- | --- | --- | --- | --- |
| K_Anat2:  All stages of swallowing are voluntary. | Anatomy and physiology | Knowledge | 0.455 | Factor loading below 0.50 |
| K_Anat6  A person can swallow and breath at the same time. | Anatomy and physiology | Knowledge | 0.252 | Factor loading below 0.50 |
| S_Scrn1  Identifying patients with possible oropharyngeal dysphagia in a timely manner can reduce the cost of treatment | Screening | Self-reported competence | 0.197 | Factor loading below 0.50 |
